# How does the food environment influence people engaged in weight management? A systematic review and thematic synthesis of the qualitative literature

**DOI:** 10.1111/obr.13398

**Published:** 2021-12-08

**Authors:** Kimberley L. Neve, Anna Isaacs

**Affiliations:** ^1^ Centre for Food Policy City, University of London London UK

**Keywords:** food environment, obesity, qualitative, weight management

## Abstract

People engaged in weight loss or weight loss maintenance (weight management) often regain weight long term. Unsupportive food environments are one of the myriad challenges people face when working towards a healthier weight. This systematic review explores how the food environment influences people engaged in weight management and the policy implications. Nine electronic databases (CINAHL, Medline, PsycINFO, Academic Search Complete, Embase, Ovid Emcare, PubMed, Open Grey, and BASE) were searched systematically in May 2020 to synthesize the qualitative evidence. Eligible studies were conducted with adults (18+) in high‐income countries, available in English and published 2010–2020 with a substantial qualitative element and reference to food environments. Data were analyzed using a thematic synthesis approach. Quality assessment using the Critical Appraisal Skills Programme was undertaken. We identified 26 studies of 679 individuals reporting on weight management experiences with reference to the food environment. Limitations of the included studies included a lack of detail regarding socioeconomic status and ethnicity in many studies. The analysis revealed that food environments undermine efforts at weight management, consistently making purchasing and consumption of healthier food more difficult, particularly for those on a low income. For weight management to be more successful, concurrent actions to reshape food environments are necessary.

**Abbreviations**
CASP: Critical Appraisal Skills Programme
HFSS: high in fat, salt, and/or sugar
SES: socioeconomic status
WMS: Weight Management Services

## 1 INTRODUCTION

Obesity and overweight are major risk factors for preventable morbidity and mortality worldwide,^1^ with a widening inequality in obesity rates.^2^, ^3^ Weight loss is associated with reductions in the risk of morbidity and mortality.^4^ For this reason and others, many people engage in weight loss, either individually or as part of Weight Management Services (WMS). Indeed, 38% of U.K. adults report trying to lose weight most of the time.^5^

In light of the link between excess weight and COVID‐19 severity,^6^ the U.K. government has invested £70 million in WMS in England, including digital apps, weight management groups, individual coaches, and clinical support.^7^ With research demonstrating the limited long‐term effectiveness of WMS, it is important to understand why individuals engaged in weight loss find it difficult to maintain their efforts.

People engaged in weight management often experience only short‐term maintenance of weight loss due to the myriad challenges they face.^8^, ^9^ Research has shown that over 80% of individuals who lose the desired amount of weight initially experience weight regain after 1 year, 85% after 2 years, and over 95% after 3 years.^10^ Individuals who experience weight regain often gain more weight than they lost during the dieting period.^11^

Two qualitative systematic reviews conducted by Greaves et al.^12^ and Spreckley et al.^13^ have explored the experiences and challenges of successful long‐term weight loss maintenance. Greaves et al.^12^ discuss the psychological “tension” between the behavior changes needed for weight loss maintenance and existing habits. Management of this tension requires constant effort through self‐regulation, renewing motivation and managing external influences such as temptations, social pressures and “high‐risk” situations, for example, holidays or stress. Spreckley et al.^13^ emphasize the importance of continuous monitoring and personalized, continuously evolving goal setting and the need to resist challenges.

The common theme in both reviews is that constant self‐regulation and monitoring were key aspects of weight loss or weight loss maintenance (henceforth weight management), as well as managing external influences. Indeed, the self‐regulation and monitoring were necessary to mitigate the challenges presented by these external influences, such as the “daunting” obesogenic food environment mentioned by Spreckley et al.^13^ However, this extrinsic challenge to weight management is not explored in detail. This reflects the tendency for discussions about weight in research to focus on the individual's behavior change and characteristics, such as self‐discipline or motivation, with less focus on how these characteristics are influenced and challenged by people's (food) environments as an important factor among wider drivers determining the condition of obesity.^9^

**FIGURE 1:**
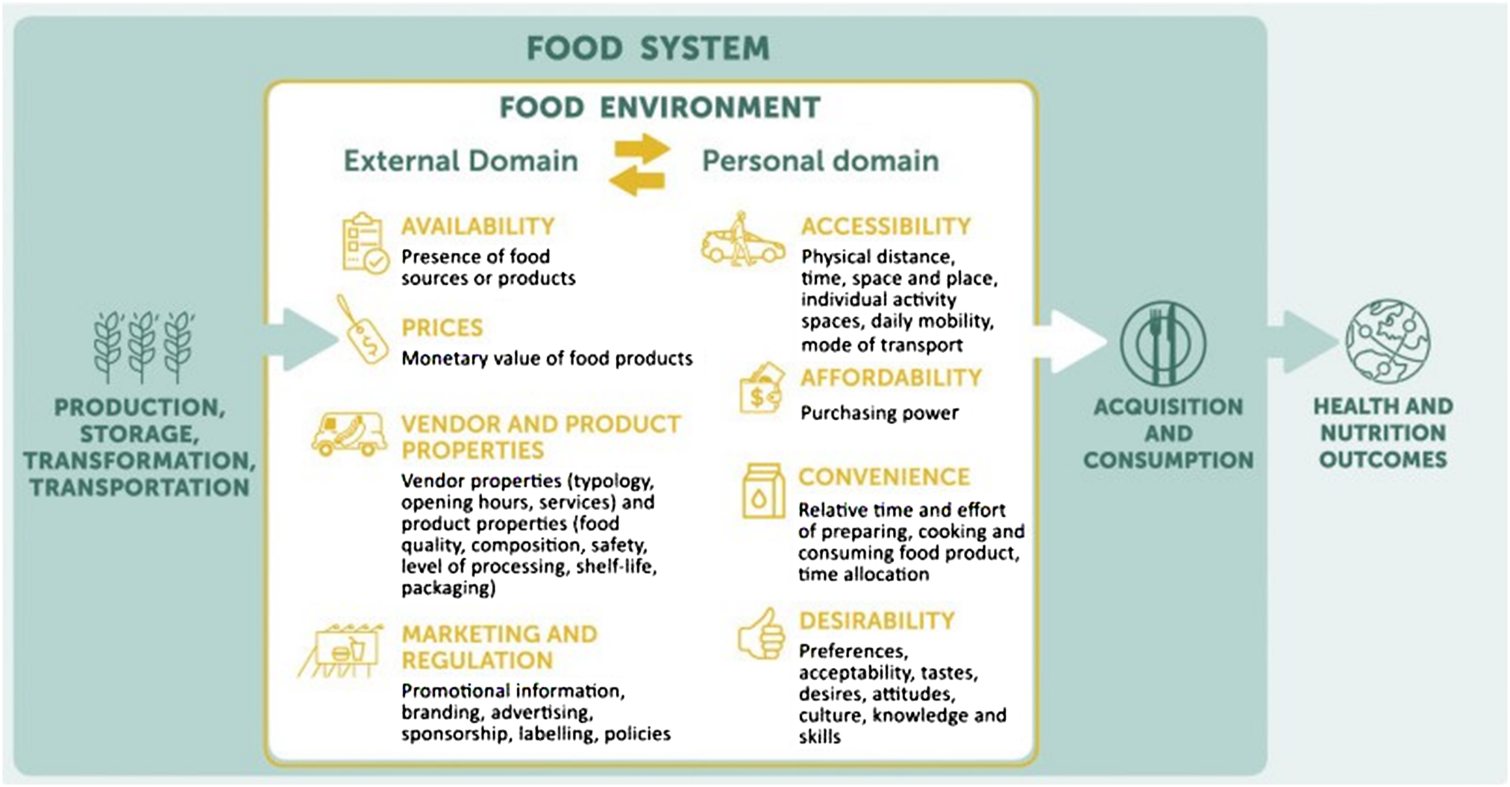
Food environments framework^18^

The term “food environment” refers to the “settings with all the different types of food made available and accessible to people in their out‐of‐home environments as they go about their daily lives”.^14^ By influencing the food and drink options people have, food environments play an important role in shaping people's diets.^15^, ^16^, ^17^ Turner et al.'s^18^ model of the Food Environment (Figure 1) shows how an individual's food environment is constructed through an interplay of personal and environmental factors. The main food environment influences on how people buy food are the availability, accessibility and affordability of food, and media and advertising.^19^ Food environments are a particular challenge for those on a low income for various reasons, which include the easy accessibility and high availability of inexpensive options high in fat, salt, and/or sugar (HFSS), particularly in low‐income areas^20^; the reduced accessibility of healthier supermarket options in some low‐income areas^21^, ^22^ and needing to go further to acquire low‐cost food^23^; and the cost of healthy food (perceived and actual).^24^

Previous qualitative research has demonstrated the challenges people face when trying to achieve a healthier weight either individually or as part of WMS^12^, ^25^ and how the food environment influences food behaviors^19^; however, the specific influence of a person's food environment on people engaged in weight management has not been explored in detail.

We undertook a systematic review of qualitative research to provide insights into (i) how people engaging in weight management experience the food environment; (ii) which aspects of the food environment impact the purchasing and consumption practices of people engaged in weight management; and (iii) what the implications are for policy to support individual weight management and WMS.

## 2 METHODS

### 2.1 Search strategy

Nine electronic databases were searched in May 2020 by KN using subject heading searches followed by keyword searches of titles and abstracts. The databases comprised CINAHL, Medline, PsycINFO, Academic Search Complete, Embase, Ovid Emcare, PubMed, Open Grey, and BASE. Terms were selected to find qualitative research papers (using non‐numerical data such as text or audio from first‐hand observation) focused on weight management experiences (people discussing their experiences of actively trying to lose weight or maintain weight previously lost). Food environment terms were not included to ensure all relevant papers that discussed weight management without these precise terms were included. A list of search terms and searching methods are provided in Tables 1 and 2.

**TABLE 1:** Search terms

Concept 1 Weight management	Concept 2 Qualitative	Exclusion criteria
Subject headings CINAHL “weight loss” OR “weight reduction programs” OR “Diet, reducing” Medline “weight loss” OR “weight reduction programs” OR “Diet, reducing” OR “obesity management” PsycInfo “weight loss” OR “weight reduction programs” OR “Diet, reducing” OR “obesity management” Academic search complete “weight loss” OR “dietary management” OR “reducing diets” Embase “body weight loss” OR “diet restriction” “weight loss program” OR “obesity management” OR “weight reduction” OR “body weight management” OR “body weight maintenance” Emcare “body weight loss” OR “diet restriction” Pubmed “obesity management” OR “weight loss” OR “weight reduction programs” OR “Diet, reducing”	Subject headings CINAHL “qualitative studies” OR “semi‐structured interview” OR “focus groups” OR “ethnological research” OR “ethnographic research” Medline “qualitative research” OR interview OR “focus groups” PsycInfo “qualitative research” OR interview OR “focus groups” OR “group discussion” Academic search complete “qualitative research” OR interviewing OR “focus groups” Embase “qualitative research” OR interview OR “ethnographic research” Emcare “qualitative research” OR interview OR “ethnographic research” Pubmed “qualitative research” OR interview as topic/methods OR “focus groups”	Published between 2010 and present Publications in English Exclude children/adolescents/infants Exclude conference abstracts Exclude clinical trials *Added for Title and Abstract screening*: Exclude those with serious/complicated/complex medical conditions, e.g. mental health, diabetes Exclude postpartum/pregnancy‐related studies Exclude if research was not conducted in a high‐income country Thesis/dissertation Feasibility studies
Keywords (obesity N3 (program* or manag* or services)) OR (weight N3 (los* or program* or reduc* or manag* or services)) OR dieting OR (diet* N3 (program* or manag* or services))	Keywords experience* OR view* OR opinion* OR preference* OR beliefs or satisf* OR qualitative OR interview* OR “focus groups” OR “group discussion” OR ethnolog* OR ethnographic OR “lived experience”	

**TABLE 2:** Search methods

Search term
Subject heading search “body weight loss” OR “diet restriction” “weight loss program” OR “obesity management” OR “weight reduction” OR “body weight management” OR “body weight maintenance”
Keyword searching (abstract) (obesity adj3 (program* or manag* or services)) OR (weight adj3 (los* or program* or reduc* or manag* or services)) OR dieting OR (diet* adj3 (program* or manag* or services))
Combine 1 and 2 with OR
Subject heading search “qualitative research” OR interview OR “ethnographic research”
Keyword searching (abstract) (experience* OR view* OR opinion* OR preference* OR beliefs or satisf* OR qualitative OR interview* OR “focus groups” OR “group discussion” OR ethnolog* OR ethnographic OR “lived experience”)
Combine 4 and 5 with OR
Combine 3 AND 6 Exclude conference abstracts Published between 2010‐present Publications in English Exclude children/adolescents/infants

### 2.2 Eligibility criteria and study selection

Articles were included if they were a primary study written in English and published between 2010 and 2020 inclusive. This time frame was chosen to ensure that participants' experiences were relevant to the present day. Studies also had to involve adults (18+) and substantial qualitative research methods, such as interviews or focus group discussions, to allow for in‐depth analysis of participant experiences. Papers could be mixed methods or wholly qualitative: If mixed methods, there needed to be a substantial qualitative element that could have stood alone as a separate qualitative study.

During title and abstract screening, studies were excluded if they involved participants with complex medical conditions, such as depression or diabetes; were postpartum and/or pregnancy‐related; or were not conducted in a high‐income country, to ensure that findings and potential policy implications were relevant to the general U.K. population. High‐income countries were defined according to the World Bank Country Classification as those with a Gross National Income of $12,696 per capita or more.^26^

The current review was explicitly interested in people's experience of losing weight or maintaining weight loss in their normal environment. Studies that focused specifically on the experience of a weight management program, rather than on weight management itself, were excluded at either the title and abstract or full‐text screening stage.

During the final paper selection, studies were excluded if there was little or no reference to the food environment due to the objectives of the study. This refinement was purposefully included later in the selection process, as it required detailed reading to determine references to the food environment that may not have used specific terminology.

### 2.3 Screening process

Articles identified through database searches were imported into EndNote version X9. Duplicate and non‐English language records were removed by KN. Two researchers (KN and AI) reviewed all titles and abstracts of the remaining articles independently using EPPI‐Reviewer 4 to identify those suitable for full‐text evaluation. Any discrepancies were discussed prior to full‐text screening.

**FIGURE 2:**
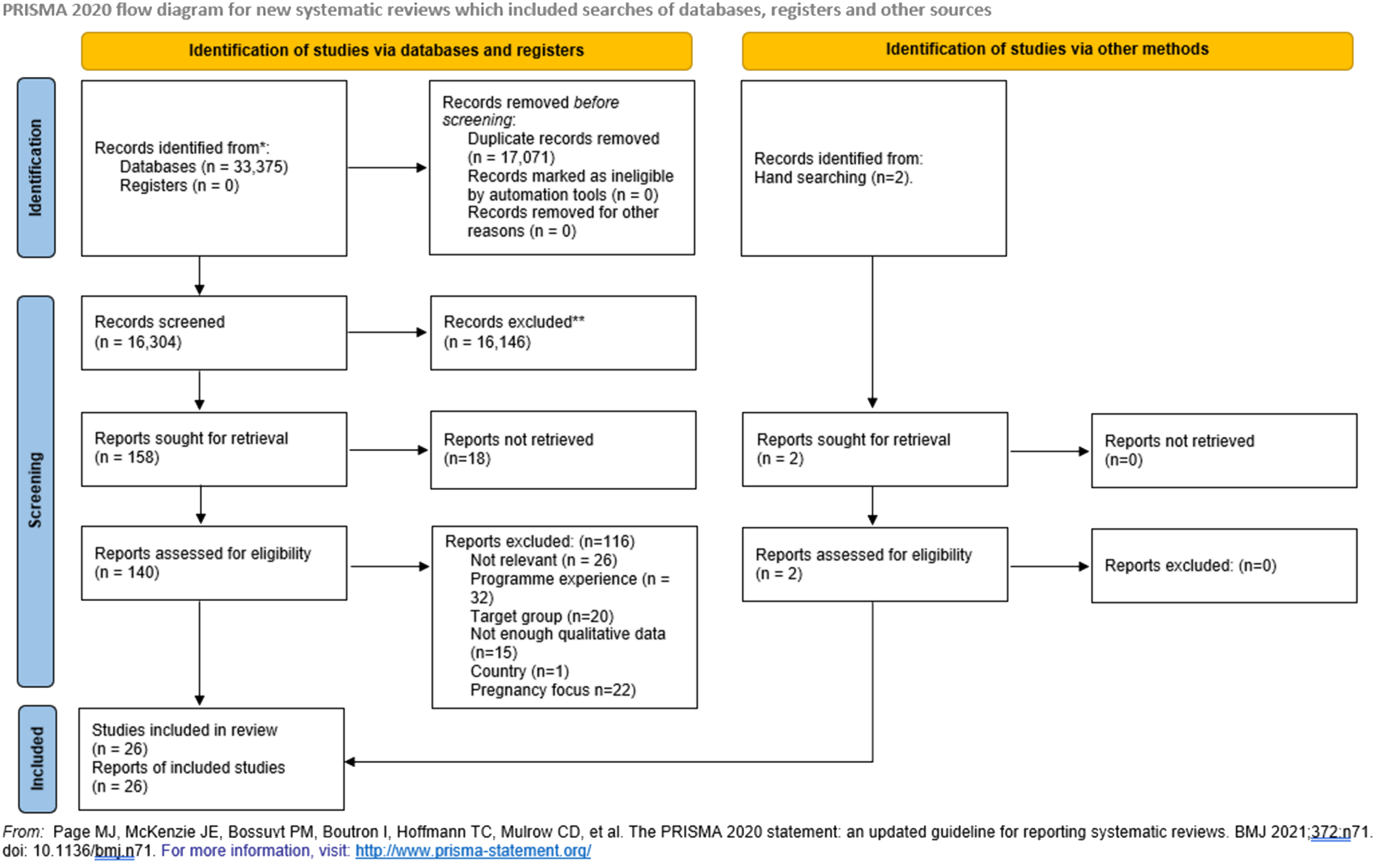
Preferred Reporting Items for Systematic Reviews and Meta‐Analyses (PRISMA) flow diagram

Inclusion or exclusion of full‐text articles was conducted independently by KN and AI, who each read all articles. Final paper selection was finalized by second readings, and any discrepancies were discussed. Two additional articles published after the search had been conducted and found independently were added. A Preferred Reporting Items for Systematic Reviews and Meta‐Analyses (PRISMA) flow diagram outlining the search and selection process can be seen in Figure 2.

### 2.4 Quality assessment

The Critical Appraisal Skills Programme (CASP) tool for appraising qualitative research was used to assess the quality of studies included in this review.^27^ No papers were excluded based on quality: The appraisal was used to understand the relative strengths and weaknesses of each paper and balance findings accordingly.

### 2.5 Coding and data analysis

Full texts were uploaded into NVIVO12 software to facilitate line by line coding. Data were analyzed following an adapted version of the thematic synthesis process outlined by Thomas and Harden.^28^ This is a widely used approach to analyzing qualitative data in a systematic review. The authors integrated stages one and two using Turner et al.'s^18^ model of the Food Environment (Figure 1) to guide the coding, rather than following an inductive coding process. This model was chosen as the basis for a deductive coding process to ensure a focused approach to analyzing the findings from the papers relevant to food environments. Turner et al.'s model considers both the physical aspects of the environment (what is available, where, etc.) and relational aspects of the food environment (the factors that might influence how people use those environments). The model divides the food environment into an external domain, composed of availability, prices, product properties, and marketing, and a personal domain comprising accessibility (defined geographically), affordability, convenience, and desirability. Although this definition of the food environment does not consider the broader systems that shape the personal domain (e.g., political and socioeconomic), or elaborate on the relationship between the personal and external factors, it is still extremely helpful in terms of thinking about the food environment's specific influence on individual actions. The researchers also allowed for elements that were not in this framework, hence the inclusion of social support as a descriptive code. Initial codes were accessibility, convenience, desirability, affordability, availability, marketing and regulation, vendor and product properties, and social support. The researchers did not code for the home food environment, as the analysis focused on the interaction between people and the out‐of‐home food environment (e.g., in supermarkets, at work or when eating out in cafes and restaurants) and its influence on food consumption.

Two researchers (KN and AI) independently coded three papers using this framework and then cross‐checked for quality assurance. The remaining articles were subsequently coded in the same manner (50% by KN; 50% by AI). The third stage was the development of analytical themes that demonstrate how the findings can be extended to generate new meaning in a different context.

## 3 RESULTS

### 3.1 Summary of included studies

A total of 33,375 papers were identified, of which 26 were included for review (Figure 2). The paucity of relevant results reflects a general lack of qualitative evidence in this area, particularly of longer‐term weight management (past the end of a specific program). Many of the qualitative studies were excluded because they focused on the experiences of a weight management program for the purpose of program evaluation, rather than the experiences and challenges of the weight management itself. Included studies were published between 2011 and 2020 from 12 high‐income countries and including the accounts of 679 individuals. None of the selected studies were COVID‐19‐related. Data collection methods included in‐depth interviews, focus group discussions, and observation. A summary of included studies can be seen in Table 3. Only six studies included data related to socioeconomic status (SES), and many studies lacked data on the ethnicity of participants. Although two studies did not specify the number of each gender, there was a clear majority of female participants across the studies.

**TABLE 3:** Summary of included studies

Author(s), year, reference	Location	Sample	Population characteristics	Data collection method	Topics studied
Abel et al. (2018)^29^	New Zealand	*n* 20 (10 males/10 females)	Varying ethnicity; SES unknown.	Interviews (*n* 20)	Facilitators and barriers that influenced the ability to make dietary improvements following a dietary intervention for pre‐diabetes.
Al‐Mohaimeed and Elmannan (2017)^30^	Saudi Arabia	*n* 37 (19 males/18 females)	Saudi ethnicity; mixed education levels.	Interviews (*n* 37)	Experiences of overweight or obesity with a particular focus on the perceived barriers and motivators to weight loss.
Bombak (2015)^31^	Canada	*n* 15 (2 males/13 females)	Minor ethnic variation (approx. 80% white).	Interviews (*n* 15) Follow‐up interviews and observation (*n* 5)	How persons with obesity, with diverse weight trajectories and views on weight loss, described their relationships to food and stigma.
Buchanan and Sheffield (2015)^32^	Australia	*n* 22 (9 males/13 females) *n* 5 (5 health professionals)	Ethnicity and SES unknown.	Focus groups (*n* 3) Focus group (*n* 1)	The phenomena of non‐adherence (diet failure) from the perspective of dieters themselves.
Clancy et al. (2018)^33^	USA	*n* 26 (1 male/25 females)	50% Black ethnicity; SES unknown.	Focus groups (*n* 8)	Specific barriers and facilitators participants faced for program engagement and weight loss. How worksites may be able to better engage employees in weight loss programs to improve their effectiveness.
Coupe et al. (2018)^34^	UK	*n* 14 (1 male/13 females)^a^	Predominantly White British, 1 Asian woman; predominantly low SES based on postcode.	Interviews (*n* 14)	Important factors to consider when tailoring lifestyle interventions for low SES populations.
Ekman (2018)^35^	Sweden	*n* 15 (5 males/10 females)	Ethnicity unclear (all born in Sweden apart from one); SES unknown.	Interviews (*n* 15)	People's own explanations of weight regain and failure to lose weight permanently. Perception of eating as a way of handling other problems and where weight reduction practices are seen as contributing to weight gain.
Eldridge et al. (2015)^36^	USA	*n* 46 (12 males/34 females)	Ethnicity—23 non‐Hispanic Black, 20 Hispanic, 3 Other; 23 unemployed, other SES details unknown.	Interviews (*n* 46)	Environmental influences on eating behavior change to promote weight loss among Black and Hispanic populations.
Hindle and Carpenter (2011)^37^	UK	*n* 10 (0 males/10 females)	Ethnicity and SES unknown.	Interviews (*n* 10)	Experiences of those who have been successful at weight maintenance.
Jackson et al. (2018)^38^	UK	*n* 15 (3 males/12 females)	73% White British; SES unknown.	Interviews (*n* 15)	Older adults' experiences of and attitudes towards weight management.
Karfopolou et al. (2013)^39^	Greece	*n* 44 (18 males/26 females)	Ethnicity and SES unknown.	Focus groups (*n* 8)	Lifestyle behaviors associated with weight regulation.
Kwasnicka et al. (2019)^40^	UK	*n* 12 (3 males/9 females)	Ethnicity and SES unknown.	Interviews (*n* 12)	Theoretical explanations of behavior change maintenance with relation to weight loss maintenance process.
Lawlor et al. (2020)^41^	UK	*n* 26 (11 males/15 females)	Predominantly White or White British (76.9%); mixed SES.	Interviews (*n* 26)	Comparison of cognitive and behavioral strategies employed to overcome “lapses” and prevent “relapse” by people who had regained weight or maintained weight loss after participating in a weight management program.
Mallyon et al. (2010)^42^	Australia	*n* 14 (8 males/6 females)	Predominantly Australian, 1 Pakistani; broadly middle class based on occupation, education and postcode.	Interviews (*n* 26)—two stages of interviews; 2 men were only interviewed once.	How men understand and practice dieting within the framework of gendered discourses and gendered relations that can make healthy eating hard to sustain.
Mastin et al. (2012)^43^	USA	*n* 46 (0 males/46 females)	African American; low‐income.	Interviews (*n* 46)	Perspectives on overweight and obesity using social cognitive theory as an interview framework.
Metzgar et al. (2015)^44^	USA	*n* 23 (0 males/23 females)	Predominantly White, 2 African American; SES unknown.	Focus groups (*n* 7)	Facilitators and barriers to weight loss and weight loss maintenance in women who previously participated in a randomized comparative trial that promoted weight loss using an energy‐restricted diet.
Natvik et al. (2018)^45^	Norway	*n* 10 (2 males/8 females)	Ethnicity and SES unknown.	Interviews (*n* 11) (1 follow‐up interview with 1 participant for further detail).	Experiences of people with severe obesity in losing weight and keeping it off for the long term.
Nielsen and Holm (2014)^46^	Denmark	*n* 25 (12 males/13 females)	Ethnicity and SES unknown.	Interviews (*n* 50) Observation of shopping events (*n* 12)	Practices and values related to food shopping for individuals who had participated in a dietary intervention.
Poltawski et al. (2020)^47^	UK	*n* 36 (14 males/22 females)	100% White ethnicity; mixed SES.	Interviews (*n* 103) 34 participants were interviewed 3 times; 34 were interviewed 2 times.	Factors that influence individual experiences and outcomes in a weight management program; why some people did better than others.
Reilly et al. (2015)^48^	Ireland	*n* 4 (male/female ratio for subset unknown)^b^	Ethnicity unknown; mixed SES.	Focus group (*n* 1)^b^	Behaviors, strategies, and attitudes associated with secondary weight maintenance and the psychological and sociocultural factors involved.
Rogerson et al. (2016)^49^	UK	*n* 8 (4 males/4 females)	White British; SES unknown.	Interviews (*n* 8)	The weight loss experiences of a sample of participants not aligned to clinical intervention research, in order to understand the weight‐loss experiences of a naturalistic sample.
Romo (2018)^50^	USA	*n* 40 (19 males/21 females)	Predominantly Caucasian/White, 13% Hispanic/Latino; mixed professions, other SES details unknown.	Interviews (*n* 40)	The communicative techniques people who have lost weight use to manage interpersonal challenges to weight management.
Sand et al. (2017)^51^	Norway	*n* 12 (0 males/12 females)	Ethnicity unknown; students, other SES details unknown.	Interviews (*n* 12)	Motivational and environmental factors that support and obstruct weight reduction and weight balance among young adult women.
Stuckey et al. (2011)^52^	USA	*n* 61 (17 males/44 females)	Ethnicity and SES unknown.	Interviews (*n 61)*	The practices and strategies that help to maintain long‐term weight loss in those who have succeeded in maintaining weight loss long term.
Yoon et al. (2018)^53^	South Korea	*n* 73 (28 males/45 females)	Ethnicity and SES unknown.	Photo‐elicitation group interviews (*n* 40) 20 groups met 2 times.	The characteristics of the food environment and its influence on weight management.
Zinn and Schofield (2012)^54^	New Zealand	*n* 25 (11 males/14 females)	Mixed ethnicities; SES unknown.	Focus groups (*n* 4)	Experiences of losing and maintaining weight and proposed ideas for a workplace‐based weight loss maintenance intervention.

^a^ This study included interviews with staff (facilitators) and attendees (service users) of a health improvement service; facilitator interviews have been excluded from this review.

^b^ Only data from participants in focus group 2—the group who had tried to lose weight and managed to sustain weight loss—were considered in this review.

### 3.2 Quality assessment

Of the 26 articles, only five met all criteria for aspects of quality within the CASP framework. However, the remaining studies predominantly lacked in the same two aspects: consideration of the relationship of the researcher to the participants and/or detail about the ethical considerations, which were likely omitted due to restrictive word limits. No papers were deemed poor quality.

### 3.3 Key findings

Four major themes were identified to represent the influence of the food environment on people engaging in weight management: (i) Constant effort is required to navigate the food environment; (ii) people's efforts are consistently undermined by the availability and accessibility of less nutritious options in food environments; (iii) higher cost (real and perceived) of healthier produce creates challenges for those on lower incomes trying to lose weight; and (iv) when social situations intersect with the food environment, weight management is particularly challenging. An overview of themes and in which studies they were mentioned can be seen in Table 4. The authors did not identify conflicting findings in the included studies.

**TABLE 4:** Overview of themes

Themes	Subthemes	References
Theme 1: Constant effort is required to navigate the food environment.	The most effective strategies for people engaging in weight management involve extensive planning around, or avoidance of, perceived unhealthy food‐provision contexts.	Abel et al. (2018), Al‐Mohaimeed and Elmannan (2017), Buchanan and Sheffield (2015), Clancy et al. (2018), Coupe et al. (2018), Eldridge et al. (2015), Hindle and Carpenter (2011), Jackson et al. (2018), Karfopolou et al. (2013), Kwasnicka et al. (2019), Lawlor et al. (2020), Mastin et al. (2012), Metzgar et al. (2015), Natvik et al. (2018), Nielsen and Holm (2014), Poltawski et al. (2020), Reilly et al. (2015), Rogerson et al. (2016), Romo (2018), Sand et al. (2017), Stuckey et al. (2011), Yoon et al. (2018)
	People have more confidence in employing effective strategies after workshop‐style education that includes a nutrition expert and some form of ongoing support.	Abel et al. (2018), Al‐Mohaimeed and Elmannan (2017), Buchanan and Sheffield (2015), Metzgar et al. (2015), Nielsen and Holm (2014), Poltawski et al. (2020), Rogerson et al. (2016), Sand et al. (2017)
Theme 2: People's efforts are consistently undermined by availability and accessibility of less nutritious options in food environments.	Food temptations are everywhere.	Al‐Mohaimeed and Elmannan (2017), Coupe et al. (2018), Ekman (2018), Jackson et al. (2018), Kwasnicka et al. (2019), Natvik et al. (2018), Poltawski et al. (2020), Yoon et al. (2018)
	Healthy options are less easily accessible and less desirable.	Al‐Mohaimeed and Elmannan (2017), Coupe et al. (2018), Kwasnicka et al. (2019), Mallyon et al. (2010), Mastin et al. (2012), Rogerson et al. (2016), Yoon et al. (2018)
	Weight management is easier when there are healthy options available.	Clancy et al. (2018), Jackson et al. (2018), Reilly et al. (2015)
Theme 3: Cost (real and perceived) of healthier produce creates challenges for those on lower incomes trying to lose weight.	Healthy foods can seem unattainable due to a higher cost (both real and perceived) than HFSS foods.	Abel et al. (2018), Coupe et al. (2018), Mastin et al. (2012), Sand et al. (2017)
	Promotions encourage spontaneity and make less nutritious options even more tempting for those on low incomes.	Ekman (2018), Natvik et al. (2018), Nielsen and Holm (2014)
Theme 4: When social situations intersect with the food environment, weight management is particularly challenging.	Social situations are difficult when trying to manage weight, as food is nearly always involved.	Bombak (2015), Jackson et al. (2018), Karfopolou et al. (2013), Lawlor et al. (2020), Mastin et al. (2012), Rogerson et al. (2016), Yoon et al. (2018), Zinn and Schofield (2012)
	How others expect someone to engage with the food environment can present a challenge to weight management strategies.	Bombak (2015), Hindle and Carpenter (2011), Kwasnicka et al. (2019), Mallyon et al. (2010), Metzgar et al. (2015), Rogerson et al. (2016), Romo (2018), Stuckey et al. (2011), Yoon et al. (2018), Zinn and Schofield (2012)

#### 3.3.1 Constant effort is required to navigate the food environment

##### The most effective strategies for people engaging in weight management involve extensive planning around, or avoidance of, perceived unhealthy food‐provision contexts

Sustained weight management requires careful navigation of work and supermarket food environments. The most effective strategies employed in the included studies involved extensive planning around, or avoidance of, perceived unhealthy food‐provision contexts and their temptations. Indeed, the importance of preparation and planning featured heavily. Challenging work food environments meant people had to employ the strategy of preparing food at home in order to avoid eating at irregular times at work,^39^, ^41^ often out of a vending machine.^43^ To avoid eating HFSS options when out of home, people engaging in weight management had to allocate extra time for preparing and cooking healthy meals from scratch.^33^, ^44^, ^45^, ^48^, ^49^, ^52^ One strategy to cope in the work environment was not carrying money so that using the vending machines was not a possibility.^47^

A variety of strategies were used to navigate supermarkets to avoid the purchasing or consumption of more than planned, including buying smaller packages, despite this being more expensive; writing shopping lists to adhere to; portioning off larger items when home; and reading labels more closely for nutrition information.^46^, ^52^ A further strategy was to avoid shopping hungry by filling up on liquids or smoking before going into a supermarket.^46^

The most effective strategies involved avoidance of the food‐provisioning environment, for example, by shopping less often^46^ or never entering certain supermarket aisles.^41^, ^46^, ^48^ In some cases, people avoided certain types of food, such as less nutritious snacks^36^ or delivery food.^39^

##### People have more confidence in employing effective strategies after workshop‐style education that includes a nutrition expert and some form of ongoing support

Informal, friendly, workshop‐style education sessions as part of WMS were viewed positively across studies as facilitators of changes in practices and sustained weight management. Practical knowledge, such as new recipes, healthy food “swaps” (where a healthier option replaces a less healthy option), portion guidance, and label reading, were valued the most.^44^, ^46^, ^47^, ^49^ People using WMS found it helpful and empowering to learn how to read labels to manage portion sizes and understand nutritional information better.^29^, ^30^ The involvement of a nutrition expert was deemed an important aspect of programs, as conflicting messages from individual research often left people confused.^32^ There was a clear consensus that it was difficult to know what is healthy or where to get reliable information, as although online blogs and social media were easy to access, people questioned their reliability.^51^

#### 3.3.2 People's efforts are consistently undermined by the availability and accessibility of less nutritious options in food environments

##### Food temptations are everywhere

People engaging in weight management reported eating more simply because food was always easily available and this close and constant exposure triggered them to want food more often.^34^, ^40^, ^45^, ^47^, ^53^ People also reported that being met everywhere with promotions made it very difficult not to think about food or make unplanned purchases of HFSS food.^35^, ^38^ Fast food marketing and advertising was seen as pervasive and a real challenge to controlling weight, as it made less nutritious options very tempting.^30^, ^53^ Fast food options were also easier to access, as they were more likely than healthier options to be located close to homes and workplaces.^34^

##### Healthier options are less easily accessible and less desirable

The limited accessibility of healthier food options was identified as a difficulty for weight management.^40^ Some people reported finding it more challenging to get to shops selling healthier foods, such as larger supermarkets, and doing so required further costs or effort for transport or delivery.^30^, ^42^ One study noted this was particularly the case in more deprived areas, where it was often necessary to take at least one bus to reach a supermarket and this necessitated taking a taxi home due to the weight of the shopping, which was an extra expense.^34^ Furthermore, participants in one study commented that both walking and taking local transport (rather than driving) meant more exposure to all the options, hence more temptation.^53^ Healthier foods were also seen as the least available ones at work.^49^

In addition, healthier options in restaurants were considered to be less tasty or satisfying and thus were less desirable than other options.^43^ People engaging in weight management saw eating out in cafes and restaurants as a time to enjoy food, which meant choosing options they knew would taste good and come in larger portion sizes.^43^

##### Weight management is easier when there are healthy options available

When healthier foods were easily available and easy to access, people engaging in weight management found it easier to follow a healthier diet.^38^ Specific examples included access to healthier options on a university campus and the ability to bring in food prepared at home^48^ and a weekly farmers' market at the workplace selling affordable boxes of fresh fruit and vegetables.^33^

#### 3.3.3 Cost (real and perceived) of healthier produce creates challenges for those on lower incomes trying to lose weight

##### Healthy foods can seem unattainable due to a higher cost (both real and perceived) than HFSS foods

A limited food budget was a significant challenge for some people engaging in weight management, with basic healthy foods seen as more expensive than less healthy options.^29^, ^34^, ^43^ Participants in one study deemed it unaffordable to have healthy foods at home^43^ and students in another study commented on the high costs of healthy food as an obstacle to buying it.^51^ When discussing the challenge of affording healthy food, study participants drew comparisons with less healthy foods, which were much more affordable.^29^, ^34^ For instance, instead of steaks, cheaper burgers that could feed more people were considered a better option.^34^ With healthy food costing more than less nutritious options, people engaging in weight management had to de‐prioritize it when managing a low budget, in particular with families.^29^, ^34^ While food was a necessity, how much money was spent on food was flexible, which meant that “food is about the first thing that suffers.”^29^

##### Promotions encourage spontaneity and make less nutritious options even more tempting for those on low incomes

When budgets are constrained, promotions have more appeal as they are perceived as a way to save money.^46^ Planned shopping lists lost relevance when faced with offers, even if foods did not match the diet plan and for some, buying the cheapest or most reduced items was a point of principle.^46^ People also reported a pleasure attached to looking for, and finding, bargains, which are most commonly for less nutritious foods and thus conflicted with trying to eat healthily.^46^ This was particularly important for people who saw themselves as “food addicts” constantly tempted by food and thus more prone to be attracted by extraordinary prices.^35^

Consequently, people on a low income had to choose between healthy eating goals and the “flexib[ility] to enjoy life in unreflected and spontaneous ways”^45^ when faced with promotions for foods that did not match their goals.

#### 3.3.4 When social situations intersect with the food environment, weight management is particularly challenging

##### Social situations are difficult when trying to manage weight, as food is nearly always involved

Socializing often means going out and eating or drinking with people, which is a challenge when following a weight management plan.^31^, ^38^, ^53^ Participants across various studies saw social situations as one of the strongest challenges, as going out acted as a “trigger” for consuming HFSS food.^39^, ^41^, ^43^, ^49^ This was the case even outside of restaurants and cafes, as friends and colleagues were likely to bring less nutritious foods to social occasions.^53^, ^54^

##### How others expect someone to engage with the food environment can present a challenge to weight management strategies

People's social networks often included people who ate the same way and disliked any change in one person's eating practices.^49^ There was thus a social pressure to eat calorie‐dense foods and drink alcohol to comply with social norms,^40^, ^53^, ^54^ with some participants eating more at social events to make others feel good.^31^, ^50^ Women often experienced pressure from other women to eat more or have a treat,^44^, ^50^ and it was common to have a “saboteur” among friends who made negative comments about weight loss or healthier eating practices.^37^ This was not restricted to women: In one study, men discussed how other men can be critical when eating in restaurants and trying to order more healthily.^42^

People used specific strategies for social occasions, such as skipping desserts and appetizers or choosing healthier options when eating in restaurants.^52^ At work and with friends, some accepted, but did not actually eat, the foods they were offered or only had a very small amount^50^; others took their own food to social occasions.^44^, ^52^ This reflects the aforementioned avoidance of perceived unhealthy food‐provisioning contexts, where social occasions involving food were also avoided or strictly navigated because social norms required the consumption of HFSS food. The expectation and pressure to eat HFSS food made social occasions uncomfortable and acted as a deterrent to social engagement or a subtle form of marginalization.^31^

## 4 DISCUSSION

This review sought to provide insights into the influence of the food environment on people engaged in weight management. Findings reflected previous research demonstrating the strategies people employ to sustain weight management and more broadly, the key aspects of the food environment that influence the food people buy and consume. Thus, individual findings were not surprising; however, it is important to have gathered this information systematically for this context. This review also goes one step further to explicitly detail how people's food environments *undermine* the strategies people employ in their efforts to manage weight. This is important, as these are people engaged in reaching and maintaining a healthy weight—they are already invested in eating a healthy diet. Yet, even for these highly motivated individuals, the food environment presents a constant challenge. For individual weight management and WMS to be more successful in future, concurrent actions to reshape food environments are vital.

People experienced the food environment in various contexts: in the supermarket, at work, when traveling from one place to another and during social occasions. Through marketing and advertising, people experienced food frequently in all contexts. For those engaged in weight management, the food environment provided constant temptation, requiring extensive planning and careful navigation to maintain a healthy diet plan. This relates to previous weight management reviews documenting the necessity to self‐regulate and manage external challenges.^12^, ^13^, ^55^ Sustained weight management often involved part or total avoidance of food‐provisioning contexts (where possible), which were rarely supportive of, or conducive to, buying healthier food options.

Individual navigation of the food environment was facilitated by knowledge and skills gained from practical, evidence‐based workshops during WMS. This helped to mitigate confusion caused by unclear labeling and misinformation online in blogs and social media. Clear nutrition information on packaging has been identified by the World Health Organization as a strategy to aid healthier food purchasing.^56^ Misinformation online falls under a newer area of research coined “digital food environments,” which “encompass the digital components that may be part of food environments and influence health and nutrition”^57^: Previous research has shown how people seeking health information online are exposed to a variety of dietary information and lifestyle advice, which often conflict with public health messages.^57^, ^58^, ^59^

Efforts to manage weight were consistently undermined by the ubiquity and desirability of HFSS options, particularly in the workplace and areas where numbers of fast food outlets were high.^20^ The importance of having healthier options more readily accessible and available in commercial areas and in workplaces has been documented previously^60^, ^61^ and was highlighted by participants in the reviewed studies. Having healthier options nearby enabled people to follow a healthy diet plan more easily when unable to prepare food in advance.

As shown in previous research, healthy food was considered to be more expensive, and HFSS food was often discounted or cheaper to start with, making it more difficult for those on a low budget to maintain a healthy diet.^19^, ^24^ With many low‐income areas more likely to have a greater number of HFSS commercial food options in the vicinity and often restricted supermarkets selling healthier food,^20^, ^21^, ^22^ this review highlights how inequitable access to healthy food could act as a barrier to effective weight management.

A key aspect of the food environment that made weight management difficult was the social aspect of eating when meeting friends or with work colleagues. Social occasions centered around food and often involved eating foods or amounts that were not planned or desired in order to meet other people's expectations, as there was a certain stigma to eating differently to others. This reflects previous research demonstrating that food and eating relate not only to health but also to pleasure and social relations.^62^

### 4.1 Implications for policy to support individual weight management and WMS

Successful weight management requires careful navigation, or avoidance, of certain parts of the existing food environment. When the incentives for eating less healthily exist everywhere, both individual attempts and well‐designed WMS will have limited impact on long‐term efforts at weight loss and weight loss maintenance. Concurrent reshaping of the food environment, in conjunction with other actions to tackle obesity as part of a whole systems approach, is necessary to help people engaged in weight management enjoy more long‐term success.^9^, ^25^ Making healthier options more easily accessible, particularly in low‐income areas, more desirable, more available, and less expensive will be the main facilitators for buying and consuming healthy foods.

Based on the findings of this review, the following areas are necessary focus points for effective policy: (i) shifting the balance so that there are more promotions and offers on healthy foods, such as fruit, vegetables and nuts, and fewer promotions and offers on HFSS foods; (ii) supporting businesses and the public sector to provide healthier options in the workplace for both lunchtimes and social occasions; (iii) providing clearer labeling on foods detailing portion sizes and nutritional information; (iv) restricting marketing on HFSS food and drink; (v) developing incentives for the introduction of more fast food outlets selling healthy options, particularly around popular work locations; and (vi) providing sustained financial support for those at the lower end of the socioeconomic spectrum to make healthy food access more equitable. Finally, WMS should recognize the significant impact of the food environment on the people they aim to support and ensure strategies around food shopping and social occasions are built into all programs.

### 4.2 Limitations and implications for further research

Although this review followed a rigorous methodology, there were some limitations to the included studies. One limitation was the general lack of specific data on SES and ethnicity in many studies, highlighting an important gap for future research. Clearer and attributable information on participants' demographic and socioeconomic situation would help put research findings into more specific contexts and has significant potential to strengthen the evidence base to inform future interventions. As the majority of studies included more women than men, more research into the male perspective of weight management would be beneficial to understand similarities and differences in experiences: One paper focused on masculinities and the experience of dieting noted a difference in how the men understood and practiced their dieting compared with the women in the study.^42^ Additionally, research tends to be published about weight loss programs; the experiences of people dieting on their own are not well‐represented in the literature.

Participants in qualitative studies are more likely to focus on barriers or facilitators at individual and social levels where they have the power to make changes and are less likely to discuss the physical food environment that they see as out of their control. Indeed, original research often did not mention the food environment specifically, or participants were not asked about the potential influence of food environment factors on their weight management. Thus, this review's wide initial search scope to not include food environments as a term to capture as many relevant perspectives as possible was a strength of the study. Future research should explore more specifically how factors of the food environment influence people engaging in weight management. This should include the digital food environment, which was identified in this review as a challenge to weight management due to the difficulty in knowing where to find reliable dietary information online. More research is needed into how conflicting and incorrect dietary information online may influence people's dietary choices and the implications for future policy.

## REGISTRATION

The current review adheres to the updated Preferred Reporting Items for Systematic Reviews and Meta‐Analyses (PRISMA) statement to ensure quality of methods^63^ and reporting and was pre‐registered (PROSPERO CRD42020183039). Search terms were refined as an amendment to the original protocol, as the searches were returning extremely large numbers of papers.

## CONFLICT OF INTEREST

There are no conflicts of interest to declare.

## AUTHOR CONTRIBUTIONS

Professor Corinna Hawkes' performed the conceptualization of the study and final review. Jessica Packer contributed advice and guidance in the initial review stages.
